# Inoculation of tomato plants with rhizobacteria suppresses development of whitefly *Bemisia tabaci* (GENNADIUS) (HEMIPTERA: ALEYRODIDAE): Agro-ecological application

**DOI:** 10.1371/journal.pone.0231496

**Published:** 2020-04-16

**Authors:** Redouan Qessaoui, Abderrahim Amarraque, Hind Lahmyed, Abdelhadi Ajerrar, El Hassan Mayad, Bouchra Chebli, Alan Stuart Walters, Rachid Bouharroud

**Affiliations:** 1 Research Unit of Integrated Crop Production, Centre Regional de la Recherche Agronomique d’Agadir, plant protection laboratory, Inezgane, Morocco; 2 Biotechnology and Environmental Engineering Team, Laboratory for Process Environmental and Energy Engineering, National School of Applied Sciences, Ibn Zohr University, Agadir, Morocco; 3 Laboratory of Biotechnologies and Valorization of Natural Resources Faculty of Sciences—Agadir, Ibn Zohr University, Agadir, Morocco; 4 Department of Plant, Soil and Agricultural Systems, College of Agricultural Sciences, Southern Illinois University, Carbondale, Illinois, United States of America; Universidade Federal de Uberlândia, BRAZIL

## Abstract

In agroecosystems, soil biodiversity is increasingly becoming more recognized as providing benefits to both plants and human health. It performs a wide variety of ecological services beyond the recycling of nutrients to plant growth and manage pests and diseases below the economic injury level. This study investigated the effects of three *Pseudomonas* isolates (Q172B, Q110B and Q036B), isolated from untreated tomato rhizospheric soil, as a biological control agent of *Bemisia tabaci* which is a key pest of tomato crops. The study was conducted under laboratory and glasshouse conditions and the water treatment was used as a control. Adult mortality rates were assessed during three days at 24h interval and larva mortality rates were evaluated during six days after treatment at 48h interval. Results indicate that Q036B isolate has a faster effect on *B*. *tabaci* adult and larvae. Under laboratory conditions, all three *Pseudomonas* isolates (Q110B, Q036B and Q172B) have a significant effect on *B*. *tabaci* adult mortality compared to control. The earliest and the most important mortality rate of 76% was recorded by Q036B. Two isolates Q036B and Q110B caused a significant mortality on *B*. *tabaci* larvae; with highest mortality effect (79%) was observed for Q036B compared to control. However, Q172B has no mortality effects on *B*. *tabaci* larvae under laboratory conditions. In glasshouse conditions, only Q036B provided high mortality rates of 91% at 168h after treatment. The results of this study indicate that the *Pseudomonas* isolate Q036B significantly suppresses *B*. *tabaci* in tomato plant and could substitute the excessive use of chemicals. Current research indicates that soil biodiversity could be promising to preserve agro-ecological sustainability.

## Introduction

Tomato (*Solanum lycopersicon L*.) is an important and one of the most widely grown vegetable crops in the world [1,2]. Recently, there has been more emphasis on tomato production, as it is not only considered as a source of vitamins, but also as a source of income and a major contributor towards food security [3]. However, there are many constraints to tomato production, including the whitefly *Bemisia tabaci* (Gennadius)(Homoptera: Aleyrodidae) [4,5], which is a vector of germinivirus like TYLCV (Tomato Yellow leaf Curl Virus) [6]. Chemical control measures have been widely utilized to manage *B*. *tabaci*, and due to their over use *B*. *tabaci* has developed resistance to some insecticides which has reduced their efficacy [5,7]. The biocontrol by use of microorganisms represents a potentially attractive alternative disease management approach [8]. For microbial biopesticides, the most commonly used is the entomopathogenic bacterium *Bacillus thuringiensis* (Bt) [9]. In addition, previous studies have reported the entomocidal activity of *B*. *thuringiensis* endotoxins to *Lygus Hesperus* knight [10], cotton aphids *Aphis gossypii and* whitefly *B*. *tabaci* [11]. However, other microorganisms have shown insecticidal properties, including *Enterobacter cloacae* that has exhibited a mild pathogenicity with 34% mortality towards the silver leaf of whitefly *B*. *argentifolii* [12].

A limited amount of research has been conducted on the insecticidal activity of *Pseudomonas*. Silva et al.[13] reported that *P*. *aeruginosa* LBI 2A1 rhamnolipids derivatives have insecticidal properties against *Aedes aegypti* larvae (Diptera: Culicidae). Concerning the mechanisms applied, Lalithambika el al.[14] indicated efficient biocontrol of *A*. *aegypti* using an exotoxin derived from *P*. *fluorescens*. Additionally, Vodovar el al.[15] reported that *P*. *entomophila* exhibited control of *Drosophila melanogaster* Meigen, which may be due to strong hemolytic activity, involving proteins such as lipases, chitinases and/or hydrolases.

The rhizospheric soil of tomatoes in Morocco is rich in bacteria. The total bacterial flora in soil was shown to be higher in samples containing roots [16]. Previous research shows a significant effect of rhizobacteria against the tomato leafminer *Tuta absoluta* and the mite *Tetranychus urticea* [17,18]. Therefore, this study investigated the efficacy of three *Pseudomonas* isolates as biological control agent, isolated from rhizospheric soil of tomatoes in Morocco, on the second instar and adults of *B*. *tabaci* under laboratory and glasshouse conditions.

## Material and methods

### *Bemisia tabaci* rearing

*B*. *tabaci* was reared on potted tomato plants *Solanum lycopersicon* (*Pristyla variety*, Gautier Seeds, France) in a glasshouse (T° = 25±2°C, RH = 70±5%) at the experimental farm of INRA (National Institute for Agricultural Research), Agadir, Morocco. The plants were grown in 5 L pots filled with a mixture of 1:2 (sterilized sand and peat). To obtain *B*. *tabaci* second instar larvae, young tomato plants (4–5 leaves) were placed in the same glasshouse close to infested plants for oviposition (48h), and then moved to another glasshouse at the same conditions (T° = 25±2°C, RH = 70±5%). It look 10 days to allow eggs to hatch and to have synchronized second instars larvae to develop on tomato [19–21].

### *Pseudomonas* isolates

The three *Pseudomonas* isolates (Q172B, Q110B, and Q036B) were isolated from an experimental farm of the INRA, Agadir, southwestern Morocco (30°02′42.2′′N 9°33′13.4′′W). The isolated bacteria were characterized in a previous work [16] based on a partial rpoD gene sequence using the primers PsrpoD FNP1 (5′-TGAAGGCGARATCGAAATCGCCAA-3′) and PsrpoDnprpcr1 (5′-YGCMGWCAGCTTYTGCTGGCA-3′) [16].

### Effects of *Pseudomonas* isolates on *B*. *tabaci*

All experiments evaluated three *Pseudomonas* isolates (Q110B, Q036B and Q172B) and a water control for influence on *B*. *tabaci* mortality.

### Laboratory bioassay on adults

Tomato leaves were collected, thoroughly washed with tap water, rewashed with sterile distilled water and then dipped for 1 min in each bacterial suspension (10^8^ cfu/mL) for each isolate. Control tomato leaves were dipped in only distilled water for 1 min. The inoculated leaves were dried and introduced individually in sterile tubes. Fifteen adult whiteflies were then introduced in tubes containing the treated tomato leaves. The tubes were sealed by muslin tissue to allow ventilation. Three replicates were evaluated for each treatment, and the bioassay was replicated thrice. The tubes were incubated at T° = 25±2°C, RH = 70±5% and adult mortality was calculated 24, 48 and 72h after treatment application.

### Laboratory bioassay on larvae

The effect of bacterial isolates on second instars *B*. *tabaci* larvae was studied under laboratory conditions using a bioassay leaf-dip technique [5,22]. A leaf cage was prepared from sterile Petri dishes (9 cm dia) containing Whatman paper soaked in sterile distilled water. 1.5 cm dia hole was made in the lid of each Petri dish and covered with muslin tissue. Tomato leaflets homogeneously infested with second instars of *B*. *tabaci* larvae were dipped in each isolate or sterile distilled water for 5s. The treated leaflets were dried under a laminar hood and then transferred to leaf cages. Four replicates for each leaf cage were used, and the experiment was replicated thrice. The cages were incubated at T° = 25±2°C, RH = 70±5% using a photoperiod of 16:8 hours (L: D). Mortality of second instars *B*. *tabaci* larvae was assessed from 24 to 168h after treatment. A larvae was considered dead once it was desiccated, and the color turned from normal pale yellow to brown [23,24].

### Glasshouse bioassay on larvae

Tomato plantlets were infested by *B*. *tabaci* adults over a five day period in a controlled climate glasshouse (T° = 25±2°C, RH = 70±5%). After egg laying, adults were removed. The same conditions were maintained for the following 12 days to allow larval development to second instar. The leaves with similar numbers of larvae (225–307 larvae) were then sprayed by each bacterial suspension (10^8^ cfu/mL) or water control using a glass hand held sprayer with a cone nozzle. After drying, clip cages (as described by Muñiz and Nombela [25]) were positioned randomly on individual leaves. Four replicates were used for each isolates and the bioassay was repeated thrice. The environmental conditions were maintained as previously mentioned (T° = 25±2°C, RH = 70±5%). The mortality rate was calculated from 24 to 168h after treatment.

### Statistical analysis

The percentage of mortality of *B*. *tabaci* larvae and adults was calculated for each *Pseudomonas* isolate. The mortality and rates, at each time, were subjected to the analysis of variance test (ANOVA) Statistica software (Version 6). Any difference mentioned is significant at p< 0.01 using the Newman–Keuls multiple range test.

## Results and discussion

### The efficacy of *Pseudomonas* isolates against *B*. *tabaci* adults

**Fig 1:**
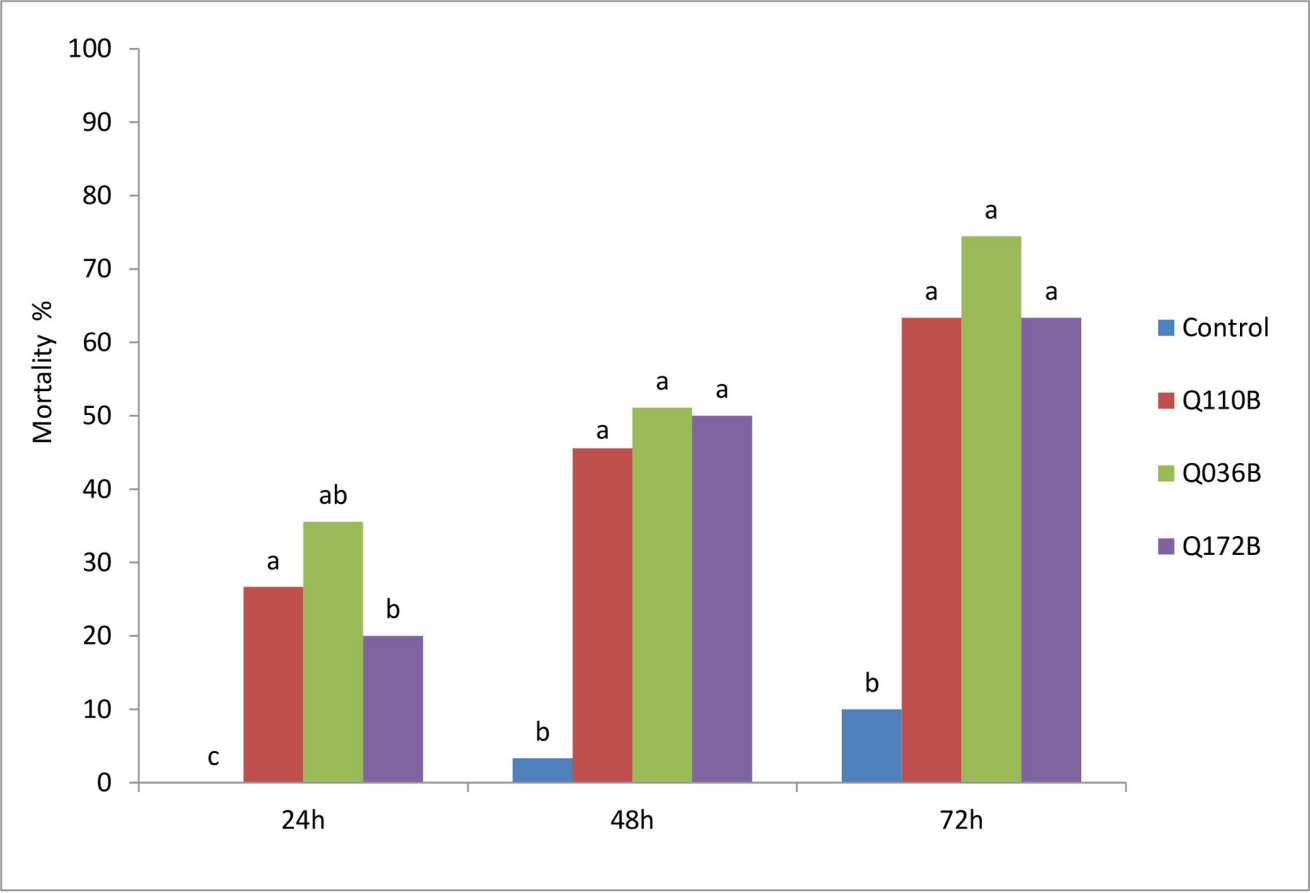
Mortality rate of *B*. *tabaci* adults over time as influenced by *Pseudomonas* isolates. Means with the same letter are not significantly according to the Newman–Keuls multiple range test (α = 0.01).

All three *Pseudomonas* isolates (Q110, Q036B and Q172B) had an effect on mortality of *B*. *tabaci* adults (Fig 1). The earliest effect of *Pseudomonas* occurred within the first day by Q036B, resulting in a 36% of mortality rate. The highest adult mortality rates was recorded 3 days after the bacterial treatment with Q036B being the highest at 76%. Adult mortality rates were higher at 24h, 48h and 72h for all *Pseudomonas* isolates compared to the control.

### Effects of *Pseudomonas* isolates on *B*. *tabaci* second instar larvae

#### Laboratory bioassay

**Fig 2:**
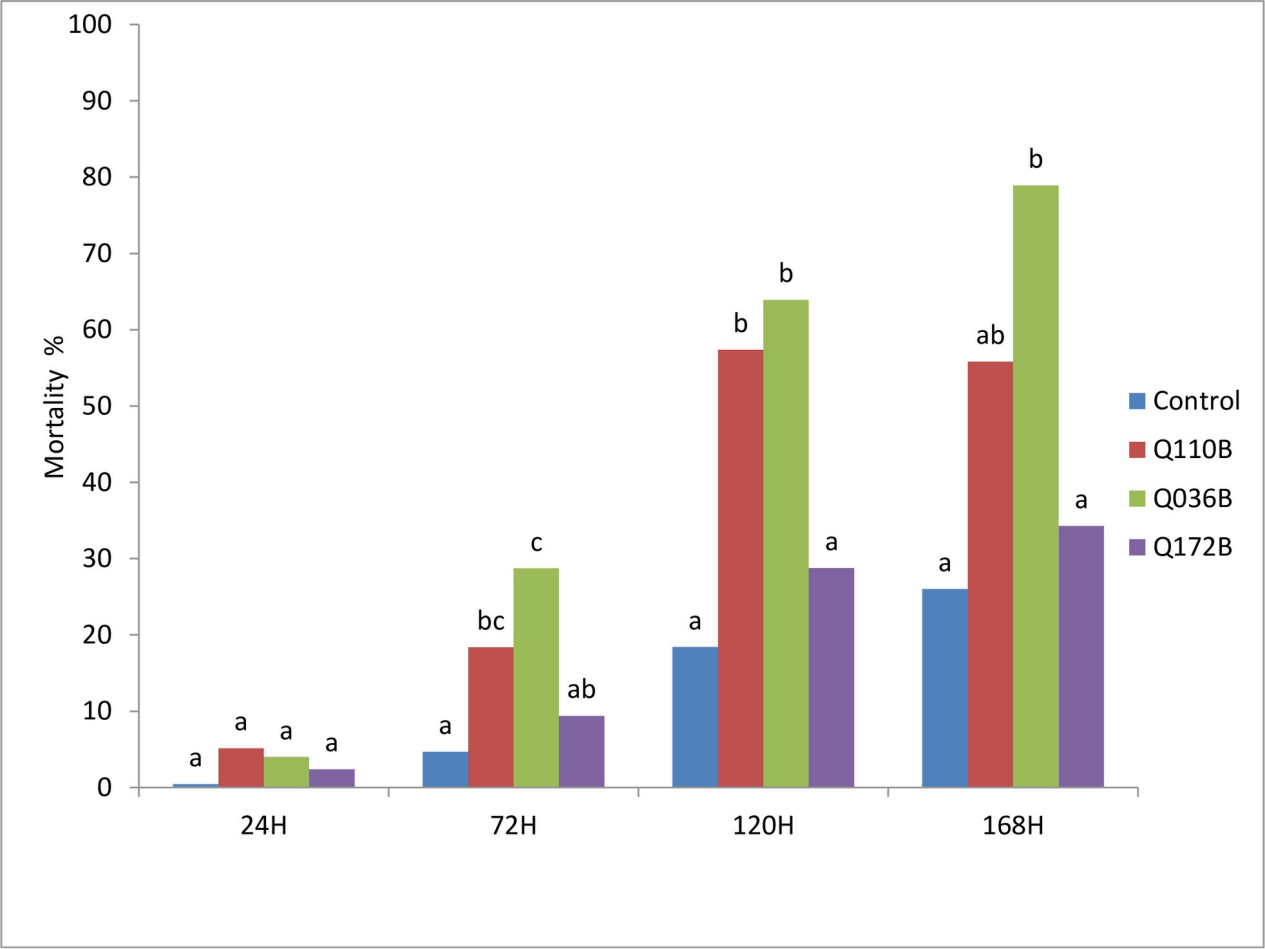
Mortality rate of *B*. *tabaci* larvae over time as affected by *Pseudomonas* isolates under laboratory conditions. Means with the same letter are not significantly different according to the Newman–Keuls multiple range test (α = 0.01).

Under laboratory conditions, the application of three *Pseudomonas* isolates on leaves infested by second instar larvae of *B*. *tabaci* provided a significant mortality (P<0.01) compared to control (Fig 2). The *Pseudomonas* isolate Q036B was the most effective and caused a 63% and 79% mortality rate at 120h and 168h after application, respectively. Another isolate Q110B provided similar high mortality rates as Q036B. However, Q172B provided no mortality to *B*. *tabaci* larvae and was similar to the water control.

#### Glasshouse bioassay

**Fig 3:**
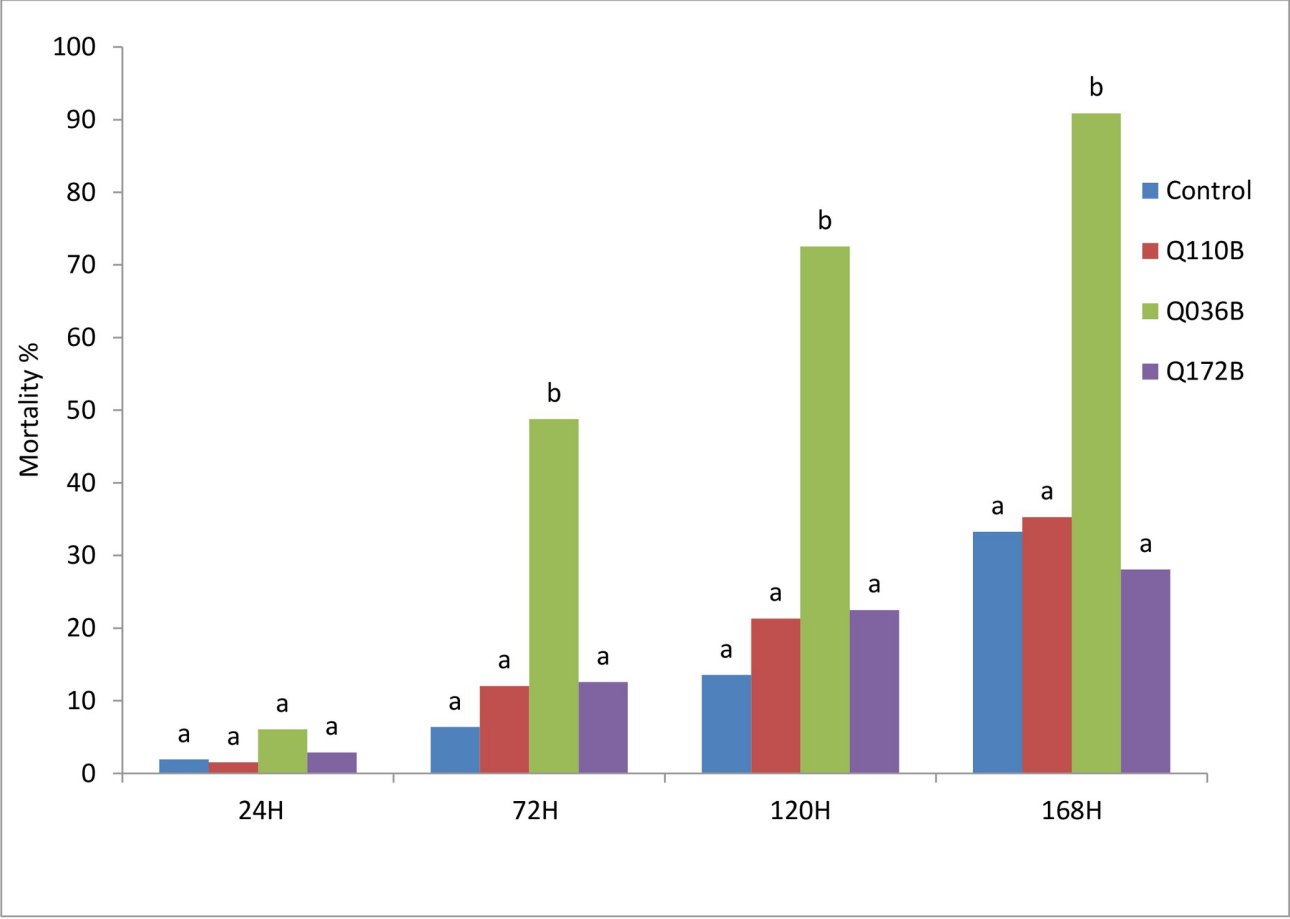
Influence of *Pseudomonas* isolate on mortalities rate of *B*. *tabaci* larvae over time under glasshouse conditions. Means with the same letter are not significantly different according to the Newman–Keuls multiple range test (α = 0.01).

The application of the three *Pseudomonas* isolates on tomato plants under glasshouse conditions provided increased mortality to *B*. *tabaci* larvae compared to the water control (P<0.01) (Fig 3).Only the Q036B isolate resulted in high mortality rate to *B*. *tabaci* larvae. All other isolates provided mortality rates similar to the water control. The Q036B isolate provided 49%, 73%, and 91% mortality to *B*. *tabaci* larva at 72h, 120h, and 168h, respectively. Moreover, no plant damage was recorded during the experiment.

## Discussion

The results of this study indicate that the three newly identified *Pseudomonas* isolates Q172B, Q110B and Q036B, isolated from rhizospheric soil of tomatoes in Morocco, have a strong efficacy resulting in mortality of *B*. *tabaci* larvae and adults in laboratory and in the glasshouse conditions. These results indicate that microbial soil biodiversity plays a dual role by protecting tomato plants against insects while also allowing a reduction in the application of insecticides.

There is evidence that the genus *Pseudomonas* contains virulent species against plant pests. Support for our results comes from the effects of different *Pseudomonas* strains on insect pests, either as bacterial suspensions or through other formulations [26,27]. In addition to the antibacterial, antifungal and nematode efficacy, *Pseudomonas sp*. has a great potential to cause insect larvae mortality. Lalithambika et al.[14] reported the efficient biocontrol of the dengue vector *A*. *aegypti* using an exotoxin derived from *P*. *fluorescens*. Mostakim et al.[28], and Omoya and Akinyosoye [29] also reported larvicidal activity of *P*. *aeruginosa* strains on *Anopheles arabiensis*, the main malaria vector in Nigeria, and the olive fruit fly *Bactroceraoleae* larvae (Diptera: Tephritidae). Additionally, Silva et al.[13] reported the larvicidal effect of *P*. *aeruginosa* LBI 2A1 derived rhamnolipids on *A*. *aegypti* larvae. A growing number of studies suggest that phyllosphere bacteria may represent a source of pathogens for plant-associated insects [30,31]. Stavrinides et al.[32] discovered that some strains of *Pseudomonas* cause high mortality rates after ingestion by pea aphids. This study provides evidence that three *Pseudomonas* isolates, isolated from rhizosphiric soil, increase mortality in the silverleaf whitefly, *B*. *tabaci*. These bacteria can act by several mechanisms in the biocontrol of diseases and pests. The ability to degrade chitin is considered the main mechanism involved in the control of pests [17,18]. Chitinolytic organisms such as *Pseudomonas sp*. and *Streptomyces sp*. isolated from the rhizosphere have been shown to be potential biocontrol agents [16,17,33]. Vodovar et al.[15] reported that *Pseudomonas entomophila* exhibits virulence against *D*. *melanogaster* due to strong hemolytic activity involving proteins such as lipases, chitinases and/or hydrolases.

Thus, pest management practices should include naturally occurring and introduced biocontrol agents. To achieve this, our study evaluated the biocontrol ability of three rhizobacterial isolates. Our findings further suggest that bacterial epiphytes and rhizospherics should be further explored for their potential use in insect pest management [30,34].

Biopesticides, such as those evaluated in our study, are becoming key components of integrated pest management programs, and are receiving practical attention as a means to reduce the amount of synthetic chemical products being used to control plant pests, and to protect stored products [35].

## Conclusion

The rhizospheric soil is rich in bacteria. It constitutes a reservoir of biocontrol agents. The fluorescent *Pseudomonas* were the most abundant bacteria in the rhizospheric soil. This study evaluated the effect of three new *Pseudomonas* Q172B, Q110B and Q036B, isolated from rhizospheric soil of tomatoes, on the second instar and adults of *B*. *tabaci* under laboratory and glasshouse conditions. The results of this study indicate that the three *Pseudomonas* tested have a strong efficacy resulting in mortality of *B*. *tabaci* larvae and adults in both laboratory and glasshouse conditions. This effect can be explained by the hemolytic activity involving proteins such as lipases, chitinases and/or hydrolases. The application of the soil microorganisms as bio-insecticide, in pest management programs, is efficient and could reduce the amount of chemicals. These results indicate that microbial soil biodiversity plays a several roles by protecting tomato plants against insects and then reducing the uses of pesticides. Therefore, they protect human and environment health.
